# Effectiveness and safety of Xuefu Zhuyu decoction for treating coronary heart disease angina: A systematic review and meta-analysis

**DOI:** 10.1097/MD.0000000000014708

**Published:** 2019-03-01

**Authors:** Tao Yang, Xiao Li, Ziwen Lu, Xiaowan Han, Mingjing Zhao

**Affiliations:** a Key Laboratory of Chinese Internal Medicine, Ministry of Education and Beijing; Laboratory for Integrated Traditional Chinese and Western Medical Research of Qi-Blood; b Department of Andrology; c Department of Cardiology, Dongzhimen Hospital, Beijing University of Chinese Medicine, Beijing, China.

**Keywords:** coronary heart disease angina, protocol, systematic review, Xuefu Zhuyu decoction

## Abstract

**Background:**

Coronary heart disease angina is a very common cardiovascular disease, which not only causes personal health problems, but also a serious burden on the social economy. Xuefu Zhuyu Decoction (XFZYD) has been widely adapted to clinical practice for people with coronary heart disease angina. At present, it is necessary to update the existing research, re-evaluate the effectiveness and safety of XFZYD, and provide the latest evidence for coronary heart disease angina.

**Methods and analysis:**

The purpose of this study was to search the electronic database for XFZYD in the treatment of coronary heart disease angina. The database includes PubMed, EMBASE, Cochrane Library, Chinese National Knowledge Infrastructure database (CNKI), Wanfang database, Chinese Biomedical Literature database (CBM), Chinese Scientific Journal database (VIP). In addition, ongoing trials will be retrieved from the WHO ICTRP Search Portal, the Chinese Clinical Trial Register and The Clinical Trials Register. We will assess all the documents from the database establishment to January 31, 2019. The RevMan V.5.3 software will be used to calculate the data synthesis and perform a meta-analysis when the literature is appropriate.

**Results:**

The study will provide a high-quality synthesis of current evidence of XFZYD for coronary heart disease angina from the various comprehensive assessment, including Significantly effective, Effective, Invalid, Aggravation, which based on the “Guidelines for Clinical Research of New Chinese Medicine”. Adverse events are also included.

**Conclusion:**

The systematic review will provide evidence for assessing the effectiveness and safety of XFZYD in the treatment of coronary heart disease angina.

**PROSPERO registration number:**

PROSPERO CRD42019122003.

## Abstract 2

Supplemental Digital Content is available in the text

## 1 Introduction

Cardiovascular disease (CVD) is a type of disease involving the heart or blood vessels, including coronary artery disease angina.^[1]^ CVD is the leading cause of death worldwide and in all regions except Africa.^[1]^ The impact of CVD on low- and middle-income countries is even higher than in high-income countries,^[2]^ CVD caused a death toll of 17.9 million (32.1%) in 2015, up from 12.3 million (25.8%) in 1990.^[3]^ It is estimated that by 2030, more than 23 million people will die of CVD each year.

Angina is usually caused by coronary arteries obstruction or spasm.^[4]^ Angina is divided into Stable angina, Unstable angina (UA), and Cardiac syndrome X (CSX).^[5]^ Traditional anti-angina medications (TAMs) include nitroglycerin, beta blockers, calcium channel blockers, angiotensin-converting enzyme inhibitors/angiotensin receptor blocker, Statins, aspirin, Ivabradine, and Lifestyle changes such as diet and exercise.^[6]^ However, TAMs do not achieve the required satisfaction due to a number of side effects such as gastrointestinal bleeding,^[7]^ decreased heart rate or blood pressure,^[8]^ and other hemodynamic changes. Therefore, it is necessary to find a method for effectively treating angina pectoris with fewer side effects.

Xuefu Zhuyu Decoction (XFZYD) was first recorded in the “Corrections on the Errors of Medical Works” in Qing Dynasty. It is a very famous Chinese medicine formula with the effect of improving microcirculation.^[9]^ The formula consists of Rehmannia root (Shengdi), Chinese Angelica (Danggui), peach seed (Taoren), Safflower (Honghua), red peony root (Chishao), hare's ear root (Chaihu), Platycodon root (Jiegeng), orange fruit (Zhiqiao), 2-toothed Achyranthes root (Niuxi), Sichuan lovage root (Chuangxiong), and prepared Liquorice root (Gancao). Pharmacological research shows that XFZYD could improve microcirculation, lowering blood lipid level, and resisting myocardial ischemia.^[10,11]^

There is no strong evidence for the effectiveness and safety of XFZYD in improving angina. Therefore, we conducted the protocol to update a systematic review for coronary heart disease angina with high-quality evidence.

## 2 Methods

The protocol has been registered on PROSPERO as CRD42019122003 (https://www.crd.york.ac.uk/prospero/display_record.php?RecordID=122003). This protocol follows the Preferred Reporting Items for Systematic Reviews and Meta-Analyses Protocols (PRISMA-P) statement guidelines. If necessary, we will describe the changes in the full review.

### 2.1 Inclusion criteria for study selection

#### 2.1.1 Types of studies

All relevant randomized controlled trials (RCTs) and semi-RCTs regarding XFZYD for the treatment of coronary heart disease angina will be included. Non-RCT, case reports and other observational studies will be excluded. No language or publication status constraints will be placed. Literature language is limited to Chinese and English.

#### 2.1.2 Types of participants

Participants, patients are 18 years of age or older and are clinically diagnosed with coronary heart disease angina according to definite diagnostic criteria by means of coronary angiography. Gender, nation, and race will not be considered.

#### 2.1.3 Types of interventions

The eligible experimental group was treated with Western medicine (WM) and XFZYD, while the control group received only the same WM.

#### 2.1.4 Types of outcome measures

##### 2.1.4.1 Primary outcomes

Main outcome indicators refer to “Guiding Principles for Clinical Research of New Chinese Medicines”.

##### 2.1.4.2 Secondary outcomes

- Mortality;
- Myocardial infarction;
- Rehospitalization rate;
- Angina pectoris Canadian Cardiovascular Society (CCS) classification;
- Adverse effects.

### 2.2 Search methods for the identification of studies

#### 2.2.1 Electronic searches

To evaluate the clinical efficacy of XFZYD in treating coronary heart disease angina, 2 members will independently search the RCTs and semi-RCTs in the following 7 Chinese and English databases, in which the data collection will form the database establishment to January 31, 2019. The databases will include PubMed, EMBASE, Cochrane Library, Chinese National Knowledge Infrastructure database (CNKI), Wanfang database, Chinese Biomedical Literature database (CBM), Chinese Scientific Journal database (VIP). Medical keywords and uncontrolled terms will be combined and retrieved in the database. Searched terms such as XFZYD, coronary heart disease, Angina Pectoris and RCT will be covered. At the same time, the original references included in the trial and the systematic review of the subject-related systematic review will serve as a supplement to the literature to ensure the recall rate. Take PubMed as an example; please refer to Appendix 1 for the search strategy.

#### 2.2.2 Searching other resources

The researchers will also manually retrieve the relevant literature, such as the WHO ICTRP Search Portal, the Chinese Clinical Trial Register and The Clinical Trials Register. What is more, we will try to acquire complete information by contacting experts in the field or corresponding author if needed.

### 2.3 Data collection and analysis

#### 2.3.1 Selection of studies

**Figure 1:**
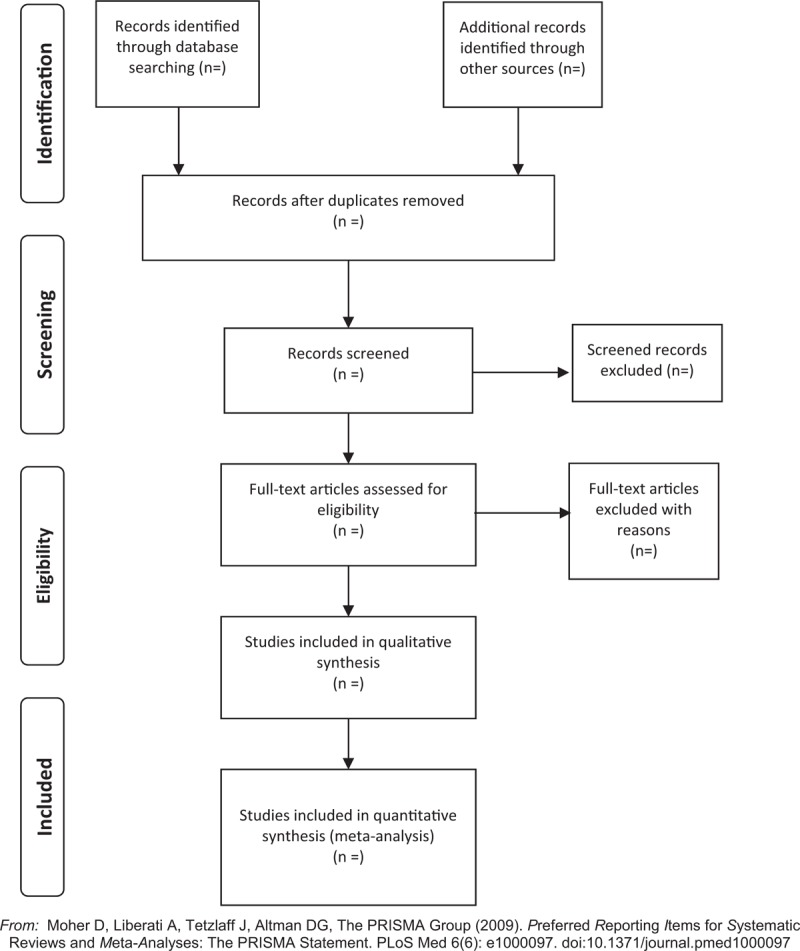
The PRISMA flow chart.

Two experienced members first searched all databases independently, screening the titles and abstracts of the relevant studies to eliminate duplication. Two additional independent researchers reviewed all extracted headlines and abstracts and screened the full text based on previous inclusion/exclusion criteria to determine eligible studies. The reasons for the excluded literature after the review will be recorded. Any disagreement will be resolved through in-group discussions or by consulting a third researcher. Eligible paper selection processes are shown in the PRISMA flow chart (Fig. 1).

#### 2.3.2 Data extraction and management

After identifying all the studies to be included, the 2 researchers will extract the following information: including but not limited to age, gender, sample size, diagnosis, intervention, and specific treatments used in the control group, course of disease, comorbidities, outcome indicators, and adverse events, and so on. Any divided opinions between reviewers will be referred to a third reviewer for arbitration.

#### 2.3.3 Assessment of risk of bias in included studies

Cochrane Collaboration tool will be adopted to the assessment of the risk of bias.^[12]^ Two trained researchers will be independently responsible for each assessment included in the study. The main items include random sequence generation, allocation concealment, blinding method for patients/researchers and outcomes assessors, incomplete outcome data, selective reporting, and other sources of bias. The results of the assessment will be divided into 3 levels, such as “low risk”, “high risk”, or “Not clear.” If there is any disagreement in the assessment, we will reach a consensus through discussion or consultation with a third reviewer.

#### 2.3.4 Measures of treatment effect

For the dichotomous results, the extracted data will be expressed as a rate ratio (RR) and a 95% confidence interval (95% CI). For continuous data, the results will be calculated as the mean difference (MD) with 95% CI.

#### 2.3.5 Dealing with missing data

The researcher will contact the corresponding author of the reference paper by email or other means to try to obtain the missing data. If this does not work, we’ll build an analysis on the available data.

#### 2.3.6 Assessment of heterogeneity

The heterogeneity in the trials will be used to assess the feasibility of the meta-analysis. If *I*^2^ is ≤50%, the statistical heterogeneity among trials can be ignored and the effect size is calculated using the fixed effects model. If *I*^2^ >50%, the heterogeneity in the trials will be significant, we will consider significant heterogeneity and perform a subgroup analysis to analyze the potential causes.

#### 2.3.7 Data synthesis and analysis

When suitable for meta-analysis, the data synthesis was performed using the software RevMan 5.3. If there is no statistical heterogeneity among the included studies, a fixed effects model is used for the analysis. Otherwise, the cause of heterogeneity should be further analyzed, analyzed using a random effects model, or turned to subgroup or sensitivity analysis, or only for descriptive analysis.

#### 2.3.8 Assessment of reporting bias

First, if more than 10 trials were included in the study, visual asymmetry on the funnel plot was used to determine if there was a publication bias. When less than 10 trials, the quantitative analysis of Egger test will be conducted using STATA 13.0 software.

#### 2.3.9 Subgroup analysis

If the included studies found significant heterogeneity and in the premise of sufficient qualified studies (at least 10 trials), we performed subgroup analyses based on patient age, gender, angina type, and adverse effects, and so on.

#### 2.3.10 Sensitivity analysis

Sensitivity analysis was performed on the primary outcome measures of the sufficient included trials to determine the robustness of the results, and low quality and small sample sizes of the literature would be excluded.

#### 2.3.11 Ethics and dissemination

The results of the systematic review will be published in peer-reviewed journals and published at relevant conferences. The data we will use does not include data for individual patients and therefore does not require ethical approval.

## 3 Discussion

**Figure 2:**
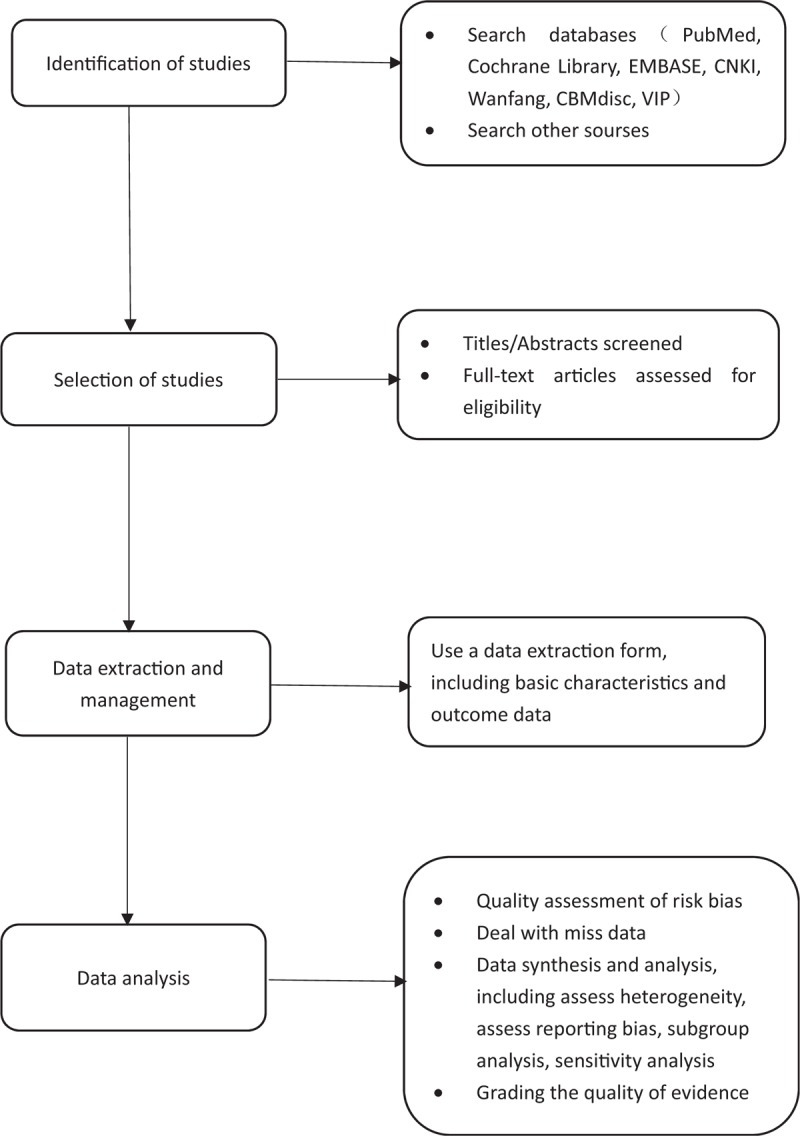
Flow diagram of the systematic review and meta-analysis.

The social and financial burden caused by coronary heart disease and angina is increasing year by year, especially in economically underdeveloped areas.^[13,14]^ Seeking safe and effective drugs or non-drug treatment has become a widespread concern in the global medical community. Clinical studies have shown that XFZYD can effectively improve the clinical symptoms of patients with coronary heart disease angina with fewer side effects,^[15,16]^ but the utility mechanism of drugs remains to be further explored. We designed and presented the latest systematic review program, using the latest data to test the effectiveness and safety of XFZYD for coronary heart disease angina. The program design is divided into 4 parts: research identification, research selection, data extraction and management, and data analysis. The process of performing this systematic review is shown in Figure 2. We hope that this study can provide more rigorous medical evidence for XFZYD in the treatment of coronary heart disease angina. However, there may still be some potential shortcomings in this study. For example, lack of high quality and large sample clinical trials, differences in dose and duration of interventions may result in significant heterogeneity. In addition, databases for search literature do not include databases from Korea and Japan and may present bias.

## Author contributions

**Data curation:** Xiao Li, Ziwen Lu.

**Formal analysis:** Tao Yang, Xiaowan Han.

**Methodology:** Tao Yang, Xiao Li.

**Project administration:** Xiao Li, Ziwen Lu.

**Software:** Tao Yang, Xiaowan Han.

**Supervision:** Mingjing Zhao.

**Validation:** Mingjing Zhao.

**Visualization:** Tao Yang, Ziwen Lu.

**Writing – original draft:** Tao Yang, Xiao Li.

**Writing – review & editing:** Mingjing Zhao.

## Supplementary Material

Supplemental Digital Content
