# Supplementary material for: Effectiveness and safety of Xuefu Zhuyu decoction for treating coronary heart disease angina: A systematic review and meta-analysis
Source: Medicine (Baltimore). 2019 Mar 1;98(9):e14708. doi: 10.1097/MD.0000000000014708 (PMC6831402; doi:10.1097/MD.0000000000014708)
Supplement: Supplemental Digital Content [file medi-98-e14708-s001.docx]

**Appendix 1. Search strategy used in PubMed database**

#1 Coronary Diseases OR Disease, Coronary OR Diseases, Coronary OR Coronary Heart Disease OR Coronary Heart Diseases OR Disease, Coronary Heart OR Diseases, Coronary Heart OR Heart Disease, Coronary OR Heart Diseases, Coronary OR Angina Pectoris OR Angina, Stable OR Angina, Unstable OR Angina Pectoris, Variant OR Microvascular Angina

#2 Xue-Fu-Zhu-Yu decoction OR [XFZY decoction OR](https://www.ncbi.nlm.nih.gov/pubmed/27473956) Xuefu Zhuyu

#3 Randomized controlled trial OR semi-randomized controlled trial OR clinical study OR Clin-ical Trial OR Controlled study OR Controlled Trial OR Random*Control* study OR random* Control* Trial

#1 AND #2 AND #3
